# Characterization of the blood oxygen level dependent hemodynamic response function in human subcortical regions with high spatiotemporal resolution

**DOI:** 10.3389/fnins.2022.1009295

**Published:** 2022-10-11

**Authors:** Jung Hwan Kim, Amanda J. Taylor, Marc Himmelbach, Gisela E. Hagberg, Klaus Scheffler, David Ress

**Affiliations:** ^1^Department of Neuroscience, Baylor College of Medicine, Houston, TX, United States; ^2^Division of Neuropsychology, Center of Neurology, Hertie-Institute for Clinical Brain Research, University of Tübingen, Tübingen, Germany; ^3^High Field Magnetic Resonance, Max Planck Institute for Biological Cybernetics, Tübingen, Germany; ^4^Department of Biomedical Magnetic Resonance, Eberhard Karl’s University of Tübingen and University Hospital, Tübingen, Germany

**Keywords:** neurovascular coupling, ultra-high field fMRI, MR magnetic field comparison, cerebral blood flow, cerebral metabolic rate of oxygen, visual cortex, superior colliculus and lateral geniculate nucleus

## Abstract

Subcortical brain regions are absolutely essential for normal human function. These phylogenetically early brain regions play critical roles in human behaviors such as the orientation of attention, arousal, and the modulation of sensory signals to cerebral cortex. Despite the critical health importance of subcortical brain regions, there has been a dearth of research on their neurovascular responses. Blood oxygen level dependent (BOLD) functional MRI (fMRI) experiments can help fill this gap in our understanding. The BOLD hemodynamic response function (HRF) evoked by brief (<4 s) neural activation is crucial for the interpretation of fMRI results because linear analysis between neural activity and the BOLD response relies on the HRF. Moreover, the HRF is a consequence of underlying local blood flow and oxygen metabolism, so characterization of the HRF enables understanding of neurovascular and neurometabolic coupling. We measured the subcortical HRF at 9.4T and 3T with high spatiotemporal resolution using protocols that enabled reliable delineation of HRFs in individual subjects. These results were compared with the HRF in visual cortex. The HRF was faster in subcortical regions than cortical regions at both field strengths. There was no significant undershoot in subcortical areas while there was a significant post-stimulus undershoot that was tightly coupled with its peak amplitude in cortex. The different BOLD temporal dynamics indicate different vascular dynamics and neurometabolic responses between cortex and subcortical nuclei.

## Introduction

Functional magnetic resonance imaging (fMRI) is a powerful tool to non-invasively quantify human brain activity. Increases in blood oxygen level-dependent (BOLD) contrast have been used as a correlate of local neural activity. Generally, such studies rely on the assumption of shift-invariant linearity between neural activity and the BOLD response, an assumption that has been partially confirmed by several experiments (Logothetis et al., 2001; Cohen et al., 2002; Heeger and Ress, 2002; Liu et al., 2010). Linear analysis relies on the BOLD hemodynamic response function (HRF), the response evoked by brief neural activation (Boynton et al., 1996; Dale and Buckner, 1997). The HRF has been extensively characterized in human cerebral cortex (Handwerker et al., 2004; Kim and Ress, 2016, 2017; Taylor et al., 2018).

Subcortical human brain regions play critical roles in functions from homeostasis to cognition. They can also be associated with cerebrovascular pathologies (e.g., traumatic brain injury, stroke) and neurodegenerative diseases (e.g., Parkinson’s and Alzheimer’s disease) (Wallace et al., 1998; Sarno et al., 2003; Burnett et al., 2004; Wedekind and Lippert-Gruner, 2005; Anderson and MacAskill, 2013; Ghose et al., 2014). Despite the critical health importance of subcortical brain regions, human subcortical research studies have been limited and mostly focused on volume reduction using structural MRI (Fearing et al., 2008; Levine et al., 2008; Yassin et al., 2015), and microstructural white matter damage using MR diffusion tensor imaging (Levine et al., 2008; Sidaros et al., 2008; Caeyenberghs et al., 2010a,b).

The anatomy of vascular perfusion in cortex has a fairly stereotypical character; pial arterioles and venules are distributed in a roughly regular pattern (Duvernoy et al., 1981). This pial mesh delivers oxygenated blood in penetrating arterioles to the gray-matter parenchyma, then deoxygenated blood drains back to the pial surface through small venules. The perfusion of brainstem nuclei, however is more variable (Tatu et al., 1998; Duvernoy, 1999). The spatial distributions of both penetrating arterioles and draining venules vary from nucleus to nucleus. For example, superior colliculus has a very regular “ladder-like” architecture of penetrating arterioles, while the lateral geniculate nucleus has a far less regular architecture. Moreover, the emergence of venous drainage from the nuclei is generally more tortuous than in cortex. Because gradient-echo BOLD contrast is believed to be dominated by venous blood oxygen changes, brainstem nuclei may therefore exhibit different neurovascular coupling from cortical regions.

It is difficult to measure the HRF in the subcortical regions because quantification of the subcortical HRF requires high spatiotemporal resolution to resolve the small nuclear subdivisions of subcortical brain regions as well as dynamics of the HRF (Singh et al., 2018). Moreover, obtaining high spatiotemporal sampling (typically ≤1.5 mm and ≤1.5 s), makes it challenging to maintain sufficient MRI signal-to-noise-ratio (SNR) because of the deep location of subcortical nuclei within the cranium and strong adjacent sources of physiological noise; SNR is typically 5–10 × lower than in cortex.

Ultra-high-field (UHF) magnetic resonance imaging (MRI) offers clear advantages for brain research studies. Early imaging problems were mostly associated with depth-of-penetration and B1-inhomogeneity issues; these issues have by now been largely remediated through use of a transmit-coil array integrated with a receive-coil array (Shajan et al., 2014). Such coil designs also enable greater acceleration factors than at low fields, especially in deeper brain regions like brainstem (Guérin et al., 2017). Functional contrast-to-noise ratio (CNR, usually defined by ratio of BOLD amplitude to its variability) appears to exhibit supra-linear increases with magnetic field strength (Uludag et al., 2009; Uludağ and Blinder, 2018; Scheffler et al., 2019). In human cerebral cortex, UHF has enabled studies with higher spatial and temporal resolution with satisfactory signal-to-noise-ratio (SNR) and CNR (Duyn, 2012; Budde et al., 2014; De Martino et al., 2018).

Temporal dynamics of the human BOLD HRF has not been well investigated. There are a few animal studies showing dynamics of the BOLD responses on subcortical regions (Yen et al., 2011; Ghodrati et al., 2017; Tong et al., 2019). However, only two previous studies have characterized the human subcortical HRF (Wall et al., 2009; Lewis et al., 2018), and these results indicate different subcortical HRFs dynamics than those in cortex. In the first, HRFs were evoked by a brief high-contrast visual stimulus and measured in visually responsive cortical and subcortical regions using conventional echo-planar imaging (EPI) at 3T with 3-mm voxels, 1.5-s sampling, and retrospective physiological noise-reduction methods (Wall et al., 2009). When averaged over subjects, their results showed that HRFs in superior colliculus (SC) were significantly different from those obtained in other brain regions. In particular, time-to-peak (TTP) was faster in SC than in early visual cortex (VC) and lateral geniculate nucleus (LGN). Despite its limited spatial resolution, this study demonstrated that subcortical HRFs are different from those evoked in cortex, which motivated further research to characterize HRFs in subcortical regions. Recently, similar significant differences of TTP among SC, LGN, and VC were found with higher spatiotemporal resolution (2-mm voxels; 1-s TR) at 7T (Lewis et al., 2018). The typical temporal dynamics of the HRF were resolved by averaging HRFs across a large pool of subjects. While the raw temporal sampling had a Nyquist frequency of 0.5 Hz, their approach required the assumption of linearity to estimate HRFs using a finite-impulse-response fitting procedure that is sensitive to CNR; regions of low CNR tend to be more heavily filtered than regions with high CNR. Thus, the precise temporal resolution of their measurements is uncertain. Moreover, the late-time dynamics of subcortical HRFs in individual subjects, such as an undershoot that is typically evident in cortex, has not been investigated. Finally, the effects of field strength on the BOLD HRF have not been sufficiently addressed.

In this study, we characterized the HRF in both subcortical areas and visual cortex using high-spatiotemporal-resolution fMRI at 9.4T and 3T. We used a slow event-related visual stimulus design that included eye-movements and a sequence-following task to activate SC, LGN, and VC simultaneously. The simple time-locked averaging approach avoided use of the assumption of linearity, so that all subjects and regions-of-interest (ROIs) were analyzed with identical temporal resolution. Temporal dynamics of the HRFs were resolved (to the Nyquist frequency of 0.4 Hz) for all individual subjects both in cortex and in subcortical nuclei at both magnetic field strengths. Timing and amplitude parameter analysis was used to examine characteristics of the subcortical HRF and its variations between cortex and subcortical nuclei. The results confirm and extend previous characterization of the subcortical HRF, providing a better understanding of neurovascular coupling in subcortical regions.

## Materials and methods

### Participants

Two different subject groups participated in experiments independently at 9.4T and 3T, with seven volunteers at each site (age 20–60 years). We expected reliable HRFs in the same region of the brain within the age range based on our previous studies demonstrating stability of the HRF across twenty subjects within a broad, sex-balanced age range of 20–60 years (Taylor et al., 2018, 2022). The volunteers for 3T experiments gave informed consent according to a protocol approved by the Baylor College of Medicine (BCM) Institutional Review Board. Our human-subjects protocol conforms to BCM’s “Ethical and Regulatory Mandate for Protecting Human Subjects,” which emphasizes the Belmont Report. To minimize effects of different scanner environments (e.g., different levels of anxiety due to different scanner hardware and field strengths), we excluded naive subjects and only recruited subjects who has been scanned multiple times. The 9.4T study was approved by the Ethics Review Board of the Eberhard Karl’s University, Tübingen and included an interview with a local physician to ascertain that all MR-safety related criteria were fulfilled. Participants provided written informed consent prior to start of the investigations, conducted in agreement with the World Medical Association (2014) Declaration of Helsinki in its most recent version.

### Stimulus

**FIGURE 1:**
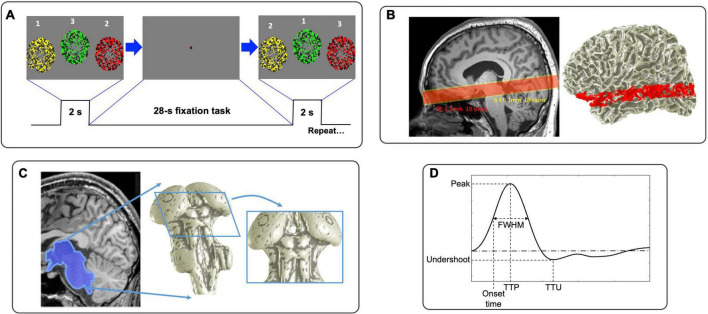
**(A)** Visual stimulus consisted of rapid sequential presentations of circular regions of flickering dots. **(B)** Functional prescriptions for 9.4T (yellow) and 3T (red). **(C)** Example of 3D segmentation of subcortical areas. **(D)** Graphical depiction of HRF parameters.

To generate brief periods of neural activity, subjects performed a visual sequence-following task every 26.25 s (25.5 s for 3T). The fixation dot changes color to cue the subject 0.5-s before a 2-s stimulation duration (Figure 1A). During this period, three circular regions (5° radius at 3T; 4° at 9.4T) filled with flickering (6 Hz), colored dots (yellow, green and red) are presented sequentially in random order. To enhance contrast, half of the dots have low saturation (“light” colors) and half have high saturation (“dark” colors). Dot color and screen position are coordinated: circular regions with yellow dots presenting on the left, green in the middle, and red on the right. Subjects were instructed to follow the sequence of flickering circular regions with eye movements and sequentially push response buttons corresponding to their color/position within 2-s stimulation duration. Thus, the task requires subject to follow a sequence of visual inputs with concurrent motor planning and response. This stimulus is followed by a 24.25-s (23.5-s for 3T) blank period to allow the subsequent HRF to evolve and decay, during which the subject performs a non-demanding, slow-paced, fixation-point color-detection task. We measured the subject’s performance by analyzing the latency and accuracy of their responses. This 26.25 s (25.5 s for 3T) duration trial is repeated 17 times in each run; 5 runs at 9.4T (8 runs at 3T) were collected. At the beginning of each run, we added a 12-s blank period to reduce the effects of MR and hemodynamic onset transients.

### Magnetic resonance imaging protocol and data preprocessing

Experiments were performed on a 9.4T Siemens MRI scanner with whole body SC 72 gradient with 80 mT/m peak and 200T/m/s slew at the Max Planck Institute (MPI) for Biological Cybernetics, Tübingen, Germany, using an in-house-built head-coil with a 16-element dual row transmit array and a 31-element receive array (Shajan et al., 2014) and a 3T Siemens Trio Scanner with 40 mT/m peak gradients and >200 T/m/s slew using 32-channel receive array at BCM, Houston, USA. At 9.4T, functional images were acquired using a point spread function (PSF) corrected EPI (In and Speck, 2012) with an inplane pixel size of 1 mm: 210 mm field of view (FOV), TE 21 ms, TR 1,250 ms, bandwidth 1254 Hz, duration of the read-out train 39.375 ms, partial Fourier in phase-encode direction of 6/8 and GRAPPA acceleration factor of 4. Functional acquisition comprised 16 quasi-axial slices (1-mm thick) (Figure 1B, yellow box). At 3T, based on the success of our previous work in the colliculi (Katyal et al., 2009; Katyal and Ress, 2014; Savjani et al., 2018; Truong et al., 2020), we used a two-shot outward-spiral acquisition (Glover, 1999; Singh et al., 2018) to obtain an inplane pixel size of 1.5 mm, 160 mm FOV with ten 1.5-mm-quasi-axial slices (Figure 1B, red box), resulting in an acquisition time of 25 ms for each shot. Considering a linear increase in signal-to-noise-ratio (SNR) with magnetic field strength, this voxel size gives similar SNR as 1 mm^3^ voxels at 9.4T. We chose TR = 750 ms with TE 35 ms, so that a volume was acquired every 1.5 s. The multiple shots were combined together by subtracting the initial value and linear trend of the phase (Pfeuffer et al., 2002); this was followed by linear trajectory correction based on a field-map collected at the start of each run (Singh et al., 2018). We prescribed slices to cover subcortical regions including SC and LGN. The slices also cover portions of early VC including V1 and V2 as well as middle temporal visual area (MT), enabling measurement of cortical HRFs for comparison.

A set of T1-weighted structural images (3D FLASH with minimum TE and TR) was obtained on the functional prescription at the beginning and end of each session: for 9.4T, 0.8 × 0.8 × 1 mm^3^ voxels, 24 slices, acquisition time (TA) 4 min; for 3T, 1 × 1 × 1.5 mm^3^ resolution, 14 slices, TA 3.5 min. These images were used to align the functional data to the segmented high-resolution structural reference volume collected in a separate session using a MP2RAGE sequence for 9.4T (0.6-mm cubic voxels with TE of 3 ms, volume TR 6,000 ms, TI 800/2,000 ms, and 5°/9° flip angle) and a MP-RAGE sequence for 3T (0.7-mm cubic voxel size, min. TE, TR 1,900 ms, TI 950 ms, and 10° flip angle).

The high-resolution volume anatomy was analyzed using the FreeSurfer software suite to segment the gray and white matter (Dale et al., 1999), with a set of “expert options” that enable segmentation at the native resolution of the data (van der Kouwe et al., 2008). From this reference anatomy, we segmented the tissue of the midbrain and portions of the thalamus using a combination of the automatic and manual methods provided by the ITK-SNAP application (Yushkevich et al., 2006). The CSF-tissue interface was then interpolated from the segmentation using isodensity surface rendering, followed by refinement using a deformable-surface algorithm based on a curvature-driven flow (Xu et al., 2006). This refined surface aids visualization of the data (Figure 1C).

The functional data was corrected for slice acquisition timing by cubic-spline interpolation after replication of initial and final time frames. Then, we compensated for head movements using motion correction with a robust intensity-based expectation-maximization algorithm (Nestares and Heeger, 2000). Next, we corrected slow baseline-intensity drift using a form of high-pass filter (Friston et al., 2000; Ress et al., 2000; Smith et al., 2004; Shmuel et al., 2007; Taylor et al., 2018, 2022; Truong et al., 2020). Specifically, a baseline was estimated from the time series by smoothing it twice using a RECT-function kernel with the same duration as HRF stimulation period, then this baseline was subtracted from the time series. Data was then transformed into the segmented reference volume using the same robust algorithm used for head motion. Thus, each volume voxel was associated with fMRI BOLD time series data. These functional imaging procedures have been previously demonstrated to provide high-quality retinotopic mapping in SC (Katyal et al., 2009; Katyal and Ress, 2014).

### Regions-of-interest

To create ROIs for SC and LGN, we used the functional data overlaid on the high-resolution volume anatomy described above. Both SC and LGN can easily be roughly located based on anatomic cues, and significant activations (CNR > 3, *p* < 0.001) were evident in the overlays. Note that CNR of 3 (3σ), corresponding to T-score of 3, indicates the confidence that the peak signal is not random noise at ∼99.7%, or *p*-value 0.003. The negative BOLD response (NBR) needs to be analyzed carefully and separately. However, there were only small portions of the NBR (<∼10%) observed in the ROIs with the given stimulus, so we excluded the NBR for further analysis by restricting the displayed activation to voxels with peak amplitude >0.2%. ROIs were then manually drawn to extract a single connected region in each SC and LGN. VC ROIs, areas V1, V2, and MT, were generated by FreeSurfer using probabilistic anatomical labeling across the cortical surface (Fischl et al., 2004). We then applied the same thresholding scheme to these ROIs. Means and standard deviations of the volumes for each ROI across subjects are 579 ± 206 mm^3^ at 9.4T (556 ± 229 mm^3^ at 3T) for SC, 615 ± 217 mm^3^ (474 ± 224 mm^3^) for LGN, 4,821 ± 2,363 mm^3^ (3,386 ± 1,432 mm^3^) for V1, 6,998 ± 829 mm^3^ (4,798 ± 1,646 mm^3^) for V2 and 2,849 ± 1,338 mm^3^ (1,453 ± 708 mm^3^) for MT.

### Hemodynamic response function analysis

Time series for each volume voxel were extracted for every 26.25 s (25.5 s for 3T) period to obtain HRFs evoked by the 2-s stimulus described above. We first averaged time series across each ROI. The trend removal in the time series preprocessing yields nearly zero-mean data. To get a more realistic estimate of the HRFs, the time series obtained from each trial was baseline adjusted by subtracting the mean of the first and last time points, so that the HRFs start from near zero amplitude. We then averaged all of repetitive HRFs in each ROI to obtain a mean HRF.

This HRF for each ROI was characterized by parameters: peak amplitude (P_*amp*_), TTP, full-width-half-maximum (FWHM), onset time and undershoot amplitude. Onset time occurs when the BOLD signal first reaches half of its peak amplitude (Figure 1D). To obtain finer parameter estimates, we upsampled each time series by a factor of 5 with cubic Hermite-spline interpolation.

The noise in fMRI data is known to have a non-Gaussian distribution (Holmes et al., 1997; Kruger and Glover, 2001). Moreover, noise distributions at 9.4T can show greater deviations from a normal distribution than at 3T because of the shorter transverse relaxation times and relatively smaller contributions from thermal noise (Triantafyllou et al., 2005; Wald and Polimeni, 2017). We therefore used a well-established resampling procedure (“bootstrapping”) to estimate the distributions of variability in BOLD contrast data obtained at 9.4T and 3T (Efron, 1987; Efron and Tibshirani, 1994). All HRFs repeats in each ROI were resampled with replacement, and then averaged. We repeated this procedure 500 times and calculated 68% confidence intervals for the HRF time samples in each ROI within a subject. For example, we collected 85 HRFs (17 event/run × 5 run) at 9.4T for each ROI. We then randomly selected with replacement from those 85 HRFs and averaged over the selected events. This process was repeated 500 times to form a bootstrapped estimate of the HRF time-series distribution. Then, we calculated mean and 68% confidence intervals for each time point of the bootstrapped HRFs. Note that this scheme implicitly accounted for multiple comparisons because it obtained the distribution upon the entire sample set. We quantified the variability as the mean difference between the upper and lower confidence intervals and the signal, equivalent to the standard-error-of-the-mean for normally distributed data. We defined CNR as the ratio of P_amp_ and its variability.

We used a similar bootstrapping scheme to estimate 68% confidence intervals for the individual time points of the mean HRF as well as distributions of HRF parameters across subjects. In each bootstrapping run, we randomly drew a HRF and its parameters with replacement from the 500 bootstrapped HRFs within each individual subject described above. Then we averaged those HRFs and parameters of all individual subjects. We repeated this procedure 2,000 times and calculated 68% confidence intervals for the HRF time samples in each ROI across subjects. To evaluate the significance of the undershoot after the hyperoxic peak, we obtained *p* values from the bootstrapped undershoot distributions across subjects, e.g., when >95% of the bootstrapped undershoot value were less than the baseline of the HRF would correspond to *p* < 0.05.

### Magnetic resonance imaging sequence and spatiotemporal resolution comparison

We used different fMRI acquisition sequences at 9.4T and 3T. At 9.4T, the PSF-corrected EPI sequence yielded good CNR in all subjects and ROIs. At 3T, a spiral acquisition was needed to provide satisfactory CNR in subcortical ROIs in all subjects. The PSF-corrected EPI sequence did not perform as well subcortically in some subjects but did yield high-CNR HRFs in VC. We performed additional experiments to measure HRFs on two subjects with both sequences at 3T. To compare the results, we normalized the measured HRFs by their peak amplitudes.

We used different voxel sizes (1-mm cubic voxels for 9.4T vs. 1.5-mm cubic voxels for 3T) and volume acquisition time (1.25 s for 9.4T, 1.5 s for 3T) because of SNR limitations at 3T. We also performed additional experiments at 9.4T on two subjects to test the effect of voxel size and volume acquisition time on the HRF. In each session, HRFs were measured in 3 runs using the same 1.5-mm voxel size and 1.5-s volume acquisition time as those at 3T. In another 3 runs, HRFs were also measured using the standard 9.4T parameters of 1-mm voxel size and 1.25-s volume acquisition. To facilitate visual comparison, these measured HRFs were normalized by their peak amplitude.

## Results

### Behavioral performance

Subjects had to push three buttons sequentially corresponding to the visual stimulus at a fast pace (667-ms for each of three circular displays), a moderately challenging task. Subjects sometimes failed to push a valid response button during a 667-ms period, but all subjects performed the task with ≥80% valid responses. Of the valid responses, accuracy varied across scans and subjects from 70 to 95%, with a mean accuracy of 83%.

### Blood-oxygen-level-dependent activations

**FIGURE 2:**
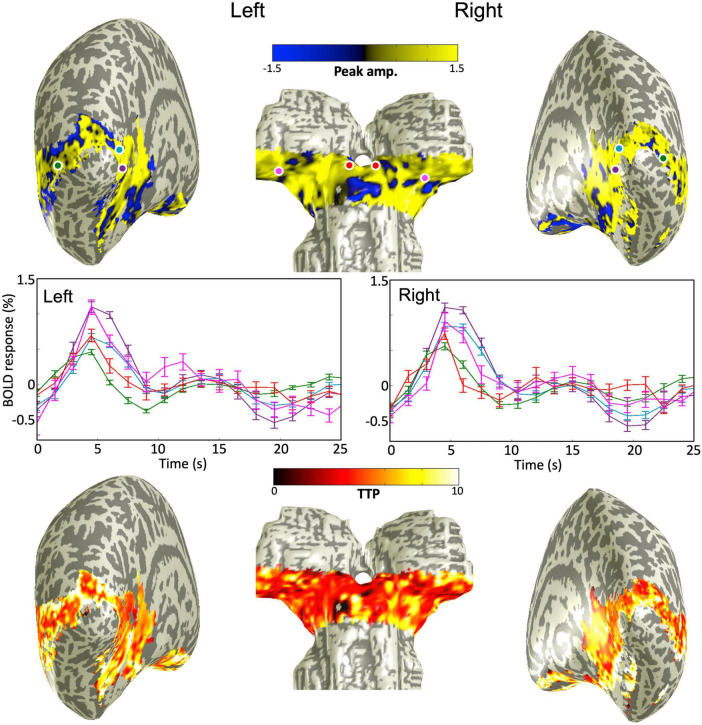
**(Upper)** peak amplitude map overlaid on smoothed gray/white matter interface mesh for one example subject at 3T. **(Middle)** Sample raw HRFs corresponding to color-coded dots on the mesh above; error bars show 68% confidence intervals. **(Bottom)** Time-to-peak map overlaid on the smoothed mesh. All maps thresholded at a CNR > 3.

The quasi-axial functional prescription covered both subcortical structures (SC and LGN) and portions of visual cortex (V1, V2, and MT). Our sequence-following task evoked strong BOLD HRFs in all these regions at both 9.4T and 3T with similar spatial activation patterns (Figure 2). Peak amplitude and TTP projected onto partially inflated cortical and subcortical surfaces for one example subject at 3T, showed broad BOLD activations across all ROIs. Sample sets of time series of the BOLD responses corresponding to color-coded dots located in the subcortical and cortical ROIs showed positive HRFs consisting of a sluggish BOLD signal increase to a hyperoxic peak and possible undershoot (and ringing—signal fluctuation after the hyperoxic peak), middle panels in Figure 2.

### Hemodynamic response function and contrast-to-noise ratio within individual subjects

**FIGURE 3:**
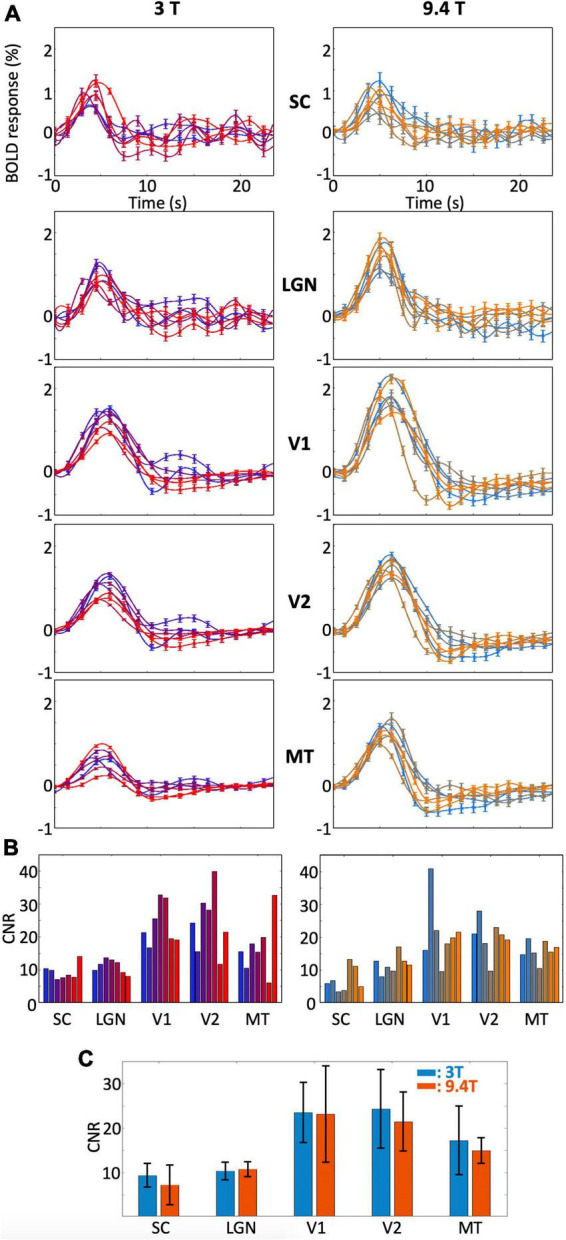
**(A)** Bootstrapped means and 68% confidence intervals of the HRF in each ROI for each subject and **(B)** CNR within all subjects. Results are shown for 3T (left) and 9.4T (right). Different colors represent different subjects. **(C)** Means and standard deviations of CNRs across subjects for each ROIs.

The peak amplitudes of the HRFs are lower in magnitude with more variability for subcortical ROIs (SC and LGN) than those for V1 and V2 within all individual subjects (Figure 3A). There was also more subject-to-subject variation of the dynamics for SC and LGN. For example, some subjects showed a fast drop from the initial peak, followed by notable ringing in SC and LGN, while other subjects showed minimal ringing. Similar variability was observed at both magnetic fields. In contrast, we found minimal ringing in all cortical ROIs. Note that the HRFs in MT showed lower peak amplitudes, similar to the HRFs in subcortical ROIs; however, the HRFs in MT were much more reliable than those in subcortical ROIs at both 9.4T and 3T. In all individual subjects, peak HRF amplitudes were reliable in both subcortical and cortical regions (*p* ≤ 0.01, peak CNR > 3).

The CNRs and their standard deviations for all individual subjects for each ROI were shown in Figure 3B. Higher CNR was found in cortical ROIs than in subcortical ROIs for both field strengths. Although peak amplitudes were overall higher at 9.4T than those at 3T, higher noise levels at 9.4T than at 3T resulted in similar CNR ranges between field strengths for all ROIs (Figure 3C).

### Comparison of hemodynamic response functions and its parameters between subcortical and cortical regions-of-interest across subjects

**FIGURE 4:**
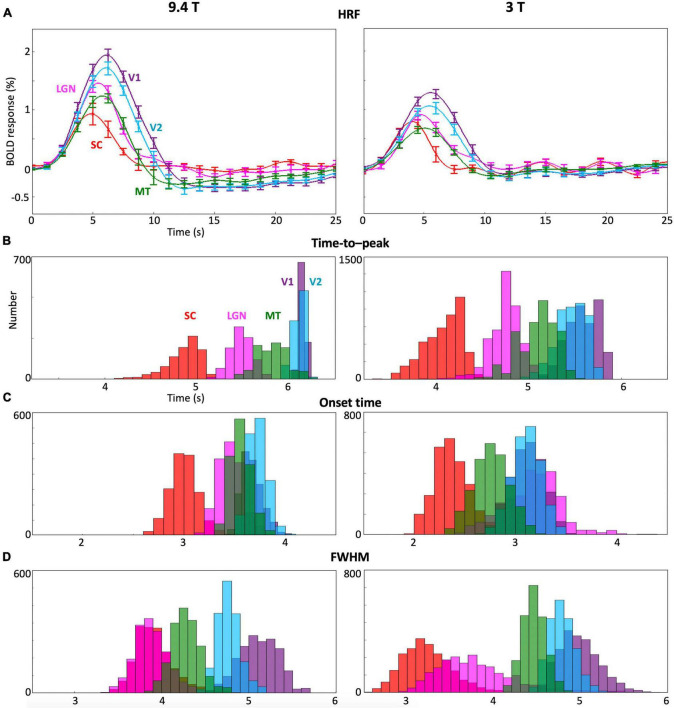
**(A)** Mean and 68% confidence intervals of the HRF across subjects for each ROI at 9.4T and 3T. **(B–D)** Bootstrapped histograms of the temporal HRF parameters across subjects in each ROI.

Cross-subject comparisons indicate that HRF dynamics were consistent across subjects in each of the subcortical and cortical ROIs (Figure 4). However, there were significant differences between ROIs. In general, HRFs in subcortical nuclei (SC and LGN) showed faster dynamics compared with early visual cortex (V1 and V2) at both 9.4T and 3T. After the hyperoxic peak, BOLD responses for the subcortical nuclei (red and magenta lines) went back to baseline with little undershoot at 9.4 T, while ringing was present at 3T. In contrast, undershoot was significant in cortical ROIs (green, light blue, and purple).

Moreover, multiple HRF temporal parameters (TTP, onset time and FHWM; Figures 4B–D) showed significant (*p* ≤ 0.01) differences between ROIs. The TTP in SC and LGN were faster than those in V1 and V2 (Figure 4B) while the TTP in SC was faster than in LGN and MT at both 9.4T and 3T. SC showed faster onset time than LGN, V1 and V2 at both 9.4T and 3T (Figure 4C). Onset time in SC was faster than in MT only at 9.4T. FHWM was narrower for subcortical nuclei (Figure 4D). At 3T, the FWHM was narrower in SC than in MT, V1, and V2. At 9.4T, SC FWHM is again narrower in SC than in V1 and V2. The FWHM also showed narrower in LGN than MT, V1, and V2. At 9.4T, narrower FWHM was observed in LGN than in V1 and V2.

### 3T vs. 9.4T hemodynamic response functions

**FIGURE 5:**
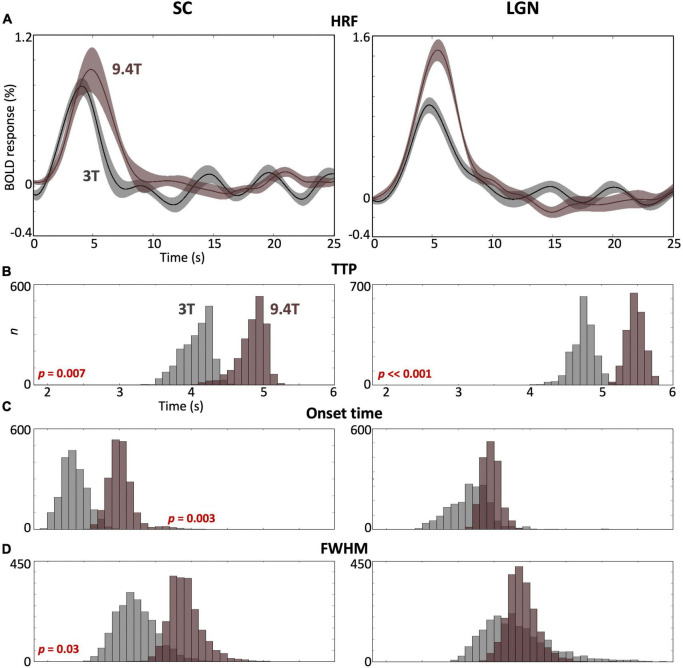
**(A)** Comparison of the mean HRF (solid) with 68% confidence intervals (shaded region) across subjects in SC and LGN between 3T (gray) and 9.4T (brown). **(B–D)** Bootstrapped histograms show HRF temporal parameter differences between field strengths with *p*-values. In SC, all parameters show significant differences at *p* ≤ 0.03. In LGN, only TTP shows a significant difference.

We observed significant differences between HRFs obtained at 3T and 9.4T for each subcortical ROI. For both SC and LGN, peak amplitudes were significantly stronger at 9.4T (brown) than at 3T (gray) (Figure 5A), while slightly greater variabilities of peak amplitude were observed at 9.4T in both ROIs. TTP values were significantly faster (*p* ≤ 0.007) at 3T than 9.4T for both ROIs (Figure 5B). SC showed significantly faster (*p* = 0.003) onset time and narrower FHWM at 3T than 9.4T, while there was no significant difference for LGN (Figures 5C,D).

**FIGURE 6:**
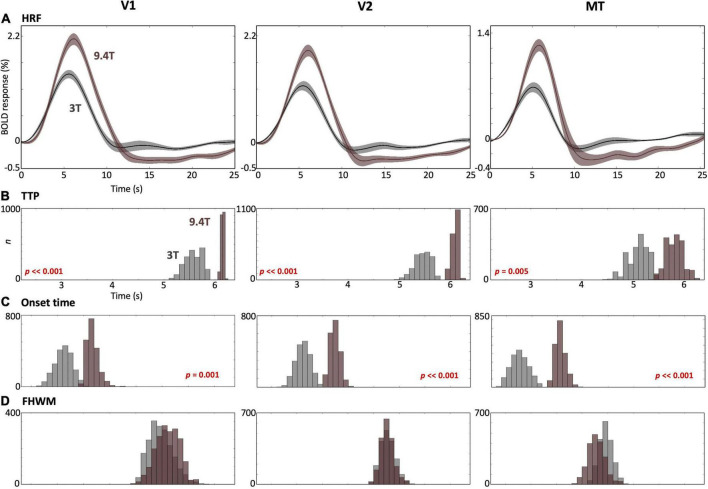
**(A)** Comparison of the mean HRF (solid) with 68% confidence intervals (shaded region) across subjects in V1 (left), V2 (middle) and MT (right) between 3T (gray) and 9.4T (brown). **(B–D)** Bootstrapped histograms below show HRF temporal parameters for both field strengths with *p*-values for significant differences.

We also found stronger peak amplitudes at 9.4T than those at 3T in all cortical ROIs (Figure 6A). The TTPs and onset times in all ROIs were significantly faster (*p* ≤ 0.005) at 3T than 9.4T (Figures 6B,C), while no significant FHWM difference was observed between field strengths (Figure 6D).

**FIGURE 7:**
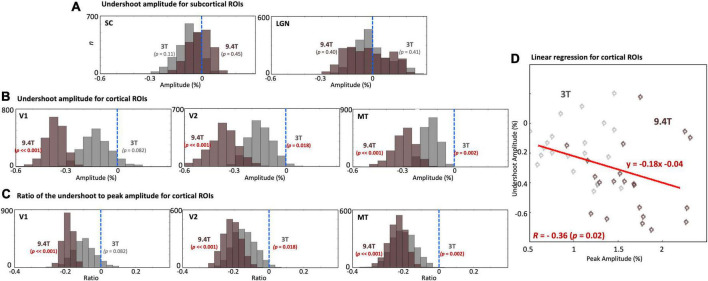
**(A)** Histograms of the undershoot amplitude across subjects in SC and LGN. No significant undershoot was found. **(B)** Histograms of the undershoot amplitude. **(C)** The ratio of the undershoot to peak amplitude across subjects in V1, V2, and MT. While significant differences of the undershoot amplitude between 9.4T and 3T were found, no significant difference was found for the ratio of the undershoot to peak amplitude between field strengths. The blue dashed vertical lines show the baseline of the HRF. **(D)** Linear regression between peak amplitude and corresponding undershoot amplitude for cortical ROIs. Each point represents mean peak and undershoot amplitudes for one subject.

We examined undershoot amplitudes in both subcortical and cortical regions. There was no significant undershoot of subcortical HRFs at both 9.4T and 3T (Figure 7A). However, there was substantial variability across subjects; a trend toward an undershoot (*p* = 0.11) was found at 3T. In contrast, significant undershoots (*p* ≤ 0.024) were found in all cortical regions (Figure 7B). Cortical undershoot amplitudes were stronger at 9.4T than those at 3T, and significantly different between field strengths in all cortical ROIs. However, there was no significant difference in the ratio of the undershoot to peak amplitude between magnetic fields strengths (Figure 7C); undershoot amplitude was correlated with corresponding hyperoxic peak amplitudes (*R* = -0.36, *p* = 0.02) at both field strengths for cortical ROIs (Figure 7D).

### Effects of MR sequences and resolution parameters

**FIGURE 8:**
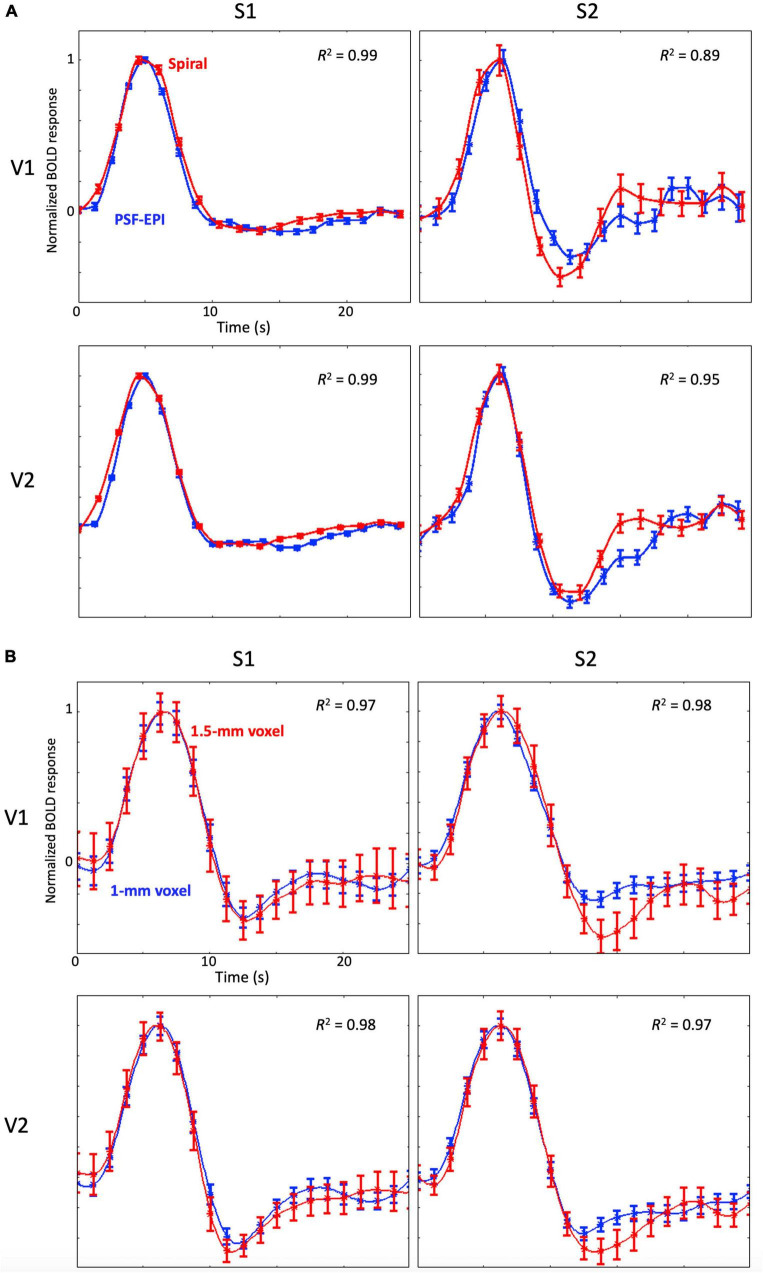
**(A)** Examples of the mean HRFs measured with the spiral sequence (red) and the PSF-EPI (blue) for two subjects at 3T; the error bars show standard error of the mean. Note that HRFs are normalized by their peak amplitude. **(B)** Examples of the mean HRFs with 1.5-mm sampling (red) and 1-mm sampling (blue) for two subjects at 9.4T; error bars show standard error of the mean. Note that HRFs are normalized by their peak amplitude.

For the comparison between spiral and PSF-EPI sequences, we observed very similar time series (*R*^2^ ≥ 0.89) without any significant differences of their temporal parameters between the two sequences, which confirms that the HRF measurements obtained by the two sequences are comparable (Figure 8A; Kim et al., 2013; Kim and Ress, 2016; Taylor et al., 2018; Truong et al., 2020).

We observed that the higher-resolution spatial and temporal sampling provided somewhat cleaner time series (Figure 8B). However, mean time series for both samplings were very similar to each other (*R*^2^ ≥ 0.97) without any significant differences between their temporal parameters.

## Discussion

We measured reliable HRFs in both cortical and subcortical regions at 9.4T and 3T in all seven subjects. The HRF was faster in subcortical regions than in cortical regions at both field strengths. In addition, the HRF in SC is faster than LGN. The undershoot at both 9.4T and 3T was only significant in cortical regions. The cortical undershoot was tightly coupled with its peak amplitude, independent of field strength.

Our visual stimulus with its sequence-following task strongly evoked positive BOLD HRFs in subcortical (SC and LGN) and cortical (V1, V2, and MT) ROIs. Subcortical human studies in these regions to investigate neurovascular coupling have been limited because of the relatively low SNR and CNR (Singh et al., 2018). Here, we successfully measured reliable HRFs in subcortical regions for all individual subjects at both 9.4T and 3T, enabling detailed characterization and comparison of the HRF between subcortical and cortical regions as well as between 9.4T and 3T.

The dynamics of BOLD responses in SC and LGN had a stereotypical form consisting of an initial delay, followed by an increase to a hyperoxic peak, and concluding with no significant undershoot when evaluated across the whole dataset. Details of the HRF dynamics were distinct between SC and LGN and also different between subcortical and cortical visual regions. For both field strengths, TTP and onset time in SC were significantly faster than those in LGN, while no significant FHWM differences were observed between them. The subcortical responses were significantly faster and narrower than responses in V1 and V2. These results indicate that distinct neurovascular coupling mechanisms exist in SC and LGN compared with visual cortex.

Different dynamics between subcortical and cortical HRFs could be the consequence of different vascular structures and density. Local changes in cerebral blood flow (CBF) will depend on the topology of the vascular network. Visual cortex has a typical cortical vascular organization: a web-like lattice of pial arterioles feeds diving arterioles of different lengths that descend into the gray-matter parenchyma with minimal branching until they rapidly bifurcate into dense mats of smaller arterioles and capillaries (Duvernoy et al., 1981; Duvernoy, 1999; Lauwers et al., 2008). SC has a somewhat different topology where a regular array of parallel penetrating arterioles dive into the tissue where they bifurcate rapidly into capillary meshes that return blood to the surface by a more tortuous venular drainage (Duvernoy, 1999). LGN has highly laminated internal vascular structure where the blood is supplied by capillaries branching out from vertical penetrating arteries from its ventral border (Fujino, 1965). These various vascular topologies could result in different temporal characteristics for the HRF with correspondingly different transport of oxygenated hemoglobin to the neuropil. Moreover, the BOLD signal in cortex is generally thought to be dominated by downstream signals from venular drainage (Turner, 2002; Kim and Ogawa, 2012), and the architecture of venous drainage is much less straightforward in subcortical nuclei than in cortex. However, subcortical HRFs are significantly faster than in cortex, perhaps suggesting that the observed HRFs are more strongly modulated by oxygen changes in more upstream vascular compartments, specifically the capillary parenchyma. Further experiments will be necessary to resolve the differential contributions of vascular compartments to the subcortical HRF.

In addition, subcortical and cortical brain regions have different neurovascular coupling dynamics that were likely the consequence of variable local oxygen metabolism (CMRO_2_) and its corresponding CBF. Such differences have been previously noted. For example, Ances et al. (2008) observed that BOLD responses were weaker in the lentiform nucleus than those observed in visual cortex for a similar change in CBF during a task-induced activation. From this result, they inferred significant differences in the ratio of CBF to CMRO_2_ between cortex and the lentiform. Another study showed significant differences for changes in BOLD, CBF, and CMRO_2_ between primate LGN and visual cortex under hyperoxia (Wibral et al., 2007). Our previous work demonstrated how the interplay of CBF and CMRO_2_ responses modulated dynamics of the cortical HRF (Kim et al., 2013; Kim and Ress, 2016). The various combination of these changes in CBF and CMRO_2_ should affect both the magnitude and temporal dynamics of the HRF among these different brain regions.

The faster dynamics in subcortical regions than visual cortex observed suggests that the dynamics of CBF and CMRO_2_ responses may be faster in subcortical nuclei than visual cortex. In particular, our results suggest that CMRO_2_ utilization occurs only during the early period of the HRF in subcortical nuclei. This is in contrast to cortex, where it has been noted that late CMRO_2_ demand could contribute to the BOLD undershoot in cortex (Masamoto and Tanishita, 2009; Vazquez et al., 2010a,b; Kim and Ress, 2016). Further experiments with simultaneous BOLD and arterial spin labeling (ASL) measurements (Huppert et al., 2006; Truong et al., 2020), combined with modeling to estimate CMRO_2_ dynamics, will be necessary to explain the temporal differences of the HRF between subcortical nuclei and visual cortex.

Our results are consistent with previous studies comparing the HRF in subcortical and cortical regions (Wall et al., 2009; Lewis et al., 2018), which also showed faster dynamics in subcortical regions. Moreover, our results extend the characterization of subcortical HRFs in three ways. First, we demonstrate methods that accurately quantify the subcortical HRF in individual subjects. All of the individual subjects showed strong and reliable HRFs at both magnetic field strengths; the CNR calculated at the HRF peak is ≥3 even at 3T. Measurements of reliable subcortical HRFs in individual subjects can provide a useful tool to understand and characterize neurovascular coupling in subcortical regions in a healthy population, which has not yet been done. Second, faster temporal dynamics in the two subcortical visual nuclei compared to cortical areas could be the consequence of processing delays along the signal path in the visual system. Previously, temporal delay of the HRF was used to understand the structure of signal lags in the white matter (Guo et al., 2022). In a similar way, our findings can be used to characterize such temporal differences. Lastly, the availability of reliable HRF measurement at 3T can motivate usage of the subcortical HRF measurement for clinical application. For example, the subcortical HRF can be used as a metric to evaluate abnormal neurovascular and neurometabolic responses. Monitoring local neurovascular and neurometabolic activities can provide more useful information on brain dysfunction, particularly for brain pathologies that affect neurovascular and neurometabolic coupling without showing structural abnormalities. Second, the higher CNR and simpler experimental paradigm enabled detailed comparisons of the late-time behavior of the HRF. Subcortical HRFs for some individual subjects showed noticeable late-time behavior—“ringing”: decaying signal fluctuation after the hyperoxic peak. In contrast, no or minimal ringing was observed in cortical ROIs. The ringing is only evident in individual subjects; it does not clearly appear on the HRFs averaged across subjects because averaging the asynchronous late-time behavior of the HRFs tends leads to cancelation of these dynamics. However, all subjects do not produce ringing. Although ringing is observed in various imaging modalities such as optical imaging spectroscopy (Martin et al., 2006), two-photon imaging (Drew et al., 2011), laser speckle (Khan et al., 2011), fMRI BOLD (Kim and Ress, 2016), and ASL (Truong et al., 2020), its existence is still controversial. In our experiment, the higher CNR with simple experimental paradigm enable characterization of early-time behavior of the HRF. However, it may not provide enough statistical power to fully resolve the weaker late-time behavior. Further subcortical HRF measurements with a larger number of subjects may resolve this issue.

Our use of high-spatiotemporal resolution enabled comparison of temporal characteristics of the HRF between different fields strengths. The HRF has been known to exhibit a stereotypical response that corresponds to the sluggish physiology of blood flow and oxygen metabolism. Moreover, with a conventional voxel (3∼4 mm), undesired signals from outside the tissue of interest (subcortical nucleus or gray matter) are inevitably introduced. With high spatial resolution (≤1.5-mm voxel), such partial volume effects are greatly reduced. Excluding such signals is more critical for the subcortical nuclei because of its weaker signal and lower SNR than in visual cortex (Singh et al., 2018). Here, we demonstrate that high spatiotemporal resolution (<1.5 s volume acquisition and <1.5 mm voxel) BOLD fMRI with depth-restricted selection of voxels only inside nuclei enables reliable measurement of HRFs in subcortical areas in individual subjects. Our results also characterized putative differences in temporal dynamics of the HRF at different magnetic field strengths.

A faster BOLD HRF was originally expected at higher field strength for various reasons. First, microvascular contributions become more strongly weighted at higher fields because of their quadratic dependence on static magnetic field, whereas large vessel contributions to the BOLD signal vary linearly with the field strength (Duong et al., 2003; Jin et al., 2006). Moreover, at UHF, the intravascular component of the BOLD response is attenuated by the short T2 of blood (Duong et al., 2003; Silvennoinen et al., 2003), which could cause a reduction of large vessel contributions to BOLD contrast. These different weightings of vascular compartments at UHF can make BOLD contrast more sensitive to the microvasculature. Finally, the convective delay for the transient cerebral blood flow response generated by upstream arterial dilation should be slightly shorter to the microvasculature than to the downstream venous vasculature. All of these effects could generate a faster BOLD HRF at UHF. However, these expectations have never been experimentally confirmed.

In fact, our results show faster temporal dynamics at 3T than at UHF. Significantly faster TTP was observed in all ROIs at 3T; onset times were also significantly faster at 3T in all ROIs except for LGN. This unexpected result needs further thought and experimentation. One possibility is that other mechanisms aside from the classical BOLD response could play a role in BOLD contrast. The classical view of BOLD contrast postulates that a single spin population undergoes a change in observed transverse relaxivity as a consequence of hemodynamic changes evoked by neural activity (Menon et al., 1993; Bandettini et al., 1994; Posse et al., 1999; Peltier and Noll, 2002). However, a variety of experiments have indicated so-called non-classical BOLD contrast behavior (Duong et al., 2003; Jin et al., 2006; Uludag et al., 2009; Kundu et al., 2012; Renvall et al., 2014). Suggested mechanisms for this non-classical behavior include chemical exchange (Kang et al., 2018) and volume effects (Speck et al., 2001; Cheng et al., 2015). In particular, our laboratory performed experiments and modeling indicating that dual spin populations contribute to BOLD contrast (Taylor et al., 2020), including a short-lived population tentatively associated with intravascular spins, and a long-lived population associated with extravascular water. Part of BOLD contrast, therefore, could be driven by volume exchange between the intravascular and extravascular volume. Many experiments indicate that such volume exchange would occur between arterial volume and extravascular fluid. Thus, early contrast at 3T could be driven by prompt inflow of brighter arterial blood displacing extravascular water, which has lower equilibrium magnetization because of its very long T1. This effect may disappear at UHF because the equilibrium magnetization of the inflowing arterial blood also becomes negligible because of its longer T1. Thus, the functional contrast creating UHF HRFs may correspond to a more purely classical BOLD response, with a time-to-peak that is not accelerated by early arterial volume-exchange effects. This is but one suggestion; further experimentation, such as multi-echo measurements, will be necessary to understand this unexpected difference between HRF dynamics at 9.4T vs. 3T.

We examined HRF peak and undershoot amplitudes in visual cortex. Stronger peak amplitude at UHF was unsurprising. However, we found that the undershoot was significantly stronger at 9.4T than 3T in early visual cortex (Figure 7B). This difference of undershoot amplitudes between field strengths disappeared after normalization with their peak amplitudes (Figure 7C). Thus, the stronger cortical undershoot can be understood as tight coupling with its peak amplitude, which is consistent with the strong correlation between peak and undershoot amplitudes across subjects observed previously (Davis, 1994; Hu et al., 1997; Siero et al., 2015; Kim and Ress, 2016; Taylor et al., 2018). This result confirms that the undershoot exists in neocortex and is linearly correlated with peak amplitude regardless of field strength. However, in subcortical regions there was no significant undershoot observed for both field strengths. This could be the consequence of the different vascular topologies that mediate the subcortical HRFs as compared to those in neocortex. Generally, the cortical HRF undershoot is associated with either a flow undershoot (Mullinger et al., 2014; Truong et al., 2020), or late increase in CMRO_2_ (Masamoto and Tanishita, 2009; Vazquez et al., 2010a,b; Kim and Ress, 2016). Accordingly, the lack of undershoot in subcortical regions suggests an absence of flow undershoot, more temporally prompt oxygen demand, or both. However, late-time subcortical HRF dynamics also showed substantial subject-to-subject variability. The observation of ringing in some subjects is consistent with an underdamped flow response, as we proposed previously (Kim et al., 2013; Kim and Ress, 2016; Taylor et al., 2018; Truong et al., 2020).

Our comparison between spiral sequence and PSF-EPI sequence at 3T for two subjects showed that there was no significant temporal difference of the HRF. Note that similar CNR between 9.4T and 3T could be mainly the consequence of effects of higher resolution at 9.4T increasing the relative contribution from thermal noise sources while physiological noise sources (Kruger and Glover, 2001; Triantafyllou et al., 2005) tend to dominate measurements at both field strengths.

There are some limitations of this study. The use of different sets of seven subjects at 9.4T and 3T is a confounding factor in our observations. However, with a similar stimulus, we previously investigated the temporal stability of the HRF with 20 healthy subjects at 3T (Taylor et al., 2018, 2022). There were small but significant variations in timing across cortex. However, the spatial pattern of these timing variations was similar across subjects, indicating stable temporal dynamics across subjects that vary modestly across different brain regions, consistent with our current results. Nevertheless, further investigation with a larger population would be desirable to confirm and extend our findings. Moreover, we excluded the NBR for analysis because of relatively small NBR regions. With the given stimulus, it is hard to quantitatively characterize the NBR. Further experiment designed for characterization of the NBR in terms of field strengths and different ROIs (subcortical/cortical regions) would provide better insights.

## Data availability statement

The raw data supporting the conclusions of this article will be made available by the authors, without undue reservation.

## Ethics statement

The studies involving human participants were reviewed and approved by Max Planck Institute for Biological Cybernetics; Tubingen; Germany (RRID:SCR_011370) Baylor College of Medicine Department of Neuroscience (RRID:SCR_007225). The patients/participants provided their written informed consent to participate in this study.

## Author contributions

JK and DR contributed to the conception, designed the experiments, and drafted the manuscript. AT and JK performed the experiments and post-data processing at 3T while GH and MH offered data collection at 9.4T. GH and KS contributed MR expertise on ultra-high field scanner. JK, AT, DR MH, and GH analyzed the data. All authors have read and approved the final manuscript.
